# CRFalign: A Sequence-Structure Alignment of Proteins Based on a Combination of HMM-HMM Comparison and Conditional Random Fields

**DOI:** 10.3390/molecules27123711

**Published:** 2022-06-09

**Authors:** Sung Jong Lee, Keehyoung Joo, Sangjin Sim, Juyong Lee, In-Ho Lee, Jooyoung Lee

**Affiliations:** 1 Basic Science Institute, Changwon National University, Changwon 51140, Korea; yeesj123@gmail.com; 2 Center for Advanced Computation, Korea Institute for Advanced Study, Seoul 02455, Korea; newton@kias.re.kr; 3 NAVER CLOVA, Seongnam 13561, Korea; dyanos@gmail.com; 4 Department of Chemistry, Kangwon National University, Chuncheon 24341, Korea; juyong.lee@kangwon.ac.kr; 5 Korea Research Institute of Standards and Science (KRISS), Daejeon 34113, Korea; ihlee@kriss.re.kr; 6 School of Computational Sciences, Korea Institute for Advanced Study, Seoul 02455, Korea

**Keywords:** protein structure prediction, sequence-structure alignment, template-based modeling, conditional random fields, boosted regression trees, CASP

## Abstract

Sequence–structure alignment for protein sequences is an important task for the template-based modeling of 3D structures of proteins. Building a reliable sequence–structure alignment is a challenging problem, especially for remote homologue target proteins. We built a method of sequence–structure alignment called CRFalign, which improves upon a base alignment model based on HMM-HMM comparison by employing pairwise conditional random fields in combination with nonlinear scoring functions of structural and sequence features. Nonlinear scoring part is implemented by a set of gradient boosted regression trees. In addition to sequence profile features, various position-dependent structural features are employed including secondary structures and solvent accessibilities. Training is performed on reference alignments at superfamily levels or twilight zone chosen from the SABmark benchmark set. We found that CRFalign method produces relative improvement in terms of average alignment accuracies for validation sets of SABmark benchmark. We also tested CRFalign on 51 sequence–structure pairs involving 15 FM target domains of CASP14, where we could see that CRFalign leads to an improvement in average modeling accuracies in these hard targets (TM-CRFalign ≃42.94%) compared with that of HHalign (TM-HHalign ≃39.05%) and also that of MRFalign (TM-MRFalign ≃36.93%). CRFalign was incorporated to our template search framework called CRFpred and was tested for a random target set of 300 target proteins consisting of Easy, Medium and Hard sets which showed a reasonable template search performance.

## 1. Introduction

Comparing a protein sequence with another sequence or a sequence with a known protein structure is one of the important tasks in bioinformatics, especially in the template-based 3D structure modeling of proteins. In spite of striking new developments in recent years (such as Alphafold [1,2]) on 3D protein structure modeling based on contact predictions via deep learning, the sequence–structure alignment method can be still useful in various stages of protein structure modeling.

In traditional template-based modeling (TBM), the model qualities are highly dependent on finding the best templates and good alignments between the target sequence and the templates. When multiple templates are given, multiple alignments between the sequence and templates [3,4,5,6] are utilized. However, multiple alignment is strongly dependent on the alignment accuracies of pair-wise sequence–sequence or sequence–structure alignments. Improving pairwise sequence–structure alignment is also important for finding better templates for protein structure modeling.

Various kinds of profile comparison methods have been developed to improve the alignment quality between sequences. For example, there are several score functions available for calculating the match scores between profiles, such as the dot product score [7], the Jensen–Shannon divergence score [8], the log average score [9] and the Pearson’s correlation score [10]. SparksX [11] builds on previous profile–profile (comparison) alignment methods of SP (SP1, SP2, SP3, SP4, SP5) series [12,13,14,15] by incorporating additional features incrementally, such as secondary structures and solvent accessibility with a linear combination. The HHpred [16], on the other hand, is based on a comparison of HMM profiles [16,17]. We note here that HHpred was not developed specifically to improve the alignment quality itself but rather mainly for efficient template search even though here in this work we compare our alignment method against HHalign in terms of modeling accuracies. More recently, more discriminative methods of conditional random fields have been applied to pairwise alignment and fold recognition. These include Contralign [18], BoostThreader [19,20] and MRFalign [21]. BoostThreader, in particular, employs a nonlinear scoring function by means of regression trees or neural networks. Recently machine learning methods based on nearest neighbor search was also applied to pairwise alignment of proteins [22]. Among these methods, the HHalign method of the HHpred has been consistently successful with particularly fast performance.

In this work, we built a method for pairwise alignment between a sequence and a structure that combines an HMM-HMM comparison scoring scheme (HHalign, HHblits [23]) and an additional nonlinear scoring function based on pairwise conditional random fields with boosted regression trees [24]. We incorporate boosted regression trees at each stage of the training steps with various features including profile–profile similarity, secondary structure similarity, similarity of the solvent accessibility, as well as environmental features. These nonlinear scoring functions are expected to provide complicated relationships between neighboring features for propensities of match states or gaps. Boosted regression trees in our CRFalign alignment models are trained on a few sets of pairwise alignments selected from SABmark benchmark set [25]. Here in this work, our main focus is on improving the sequence–structure pairwise alignment in terms of the structure modeling accuracies that entails from those alignments. As for the important area of template search [26] based on the present alignment method, we will briefly discuss our test template search results on a set of 300 targets by incorporating CRFalign into our fold recognition framework called CRFpred [27].

One distinctive feature of CRFalign is that it combines (in an additive way) HMM-HMM comparison scores and additional nonlinear scoring scheme implemented in multiple steps of boosted regression trees. That is, the additional nonlinear scoring part is constructed by a sum of residual training steps. Therefore, we expect that the HMM-HMM comparison scores guarantee a reasonable baseline performance and the additional nonlinear scoring part learns the mismatch between the true structural alignment and incorrect alignment (induced from the incorrect HMM profiles) so that it learns better how to align the sequence and structure when the HMM-HMM scores are not reliable enough. These residual learning incorporates comparison of several predicted structural features of the target sequence and the true structural features of the templates including secondary structures and solvent accessibilities such that complex relationships between environmental features can be obtained. These features can be contrasted with MRFalign [21] or ExMachina method [22] which admit only sequence profiles as input. Hence, CRFalign is expected to be relatively effective for hard targets for which close sequence homologues are not available.

We find that improvement of alignment accuracy can be achieved, especially for pairwise alignments between protein sequence and structures with remote homologues. We evaluated the alignment quality of CRFalign by modeling the 3D structures via Modeller for different test sets from SABmark benchmark database and some CASP targets [28,29]. Here, we found that the TM scores and RMSD scores of modeled structures from CRFalign showed improvement over those from base model alignments especially for the case of hard targets. The performance test of CRFalign on 51 sequence–structure pairs involving 15 hard target domains of CASP14 and CRFalign resulted in average modeling accuracies of TM-CRFalign ≃42.94% compared with that of HHalign, which shows TM-HHalign ≃39.05% and also with that of MRFalign showing TM-MRFalign ≃36.93%. As mentioned above, we also performed a test of template search using CRFalign method on a set of 300 targets, which showed a reasonable performance.

## 2. Materials and Methods

**Figure 1:**
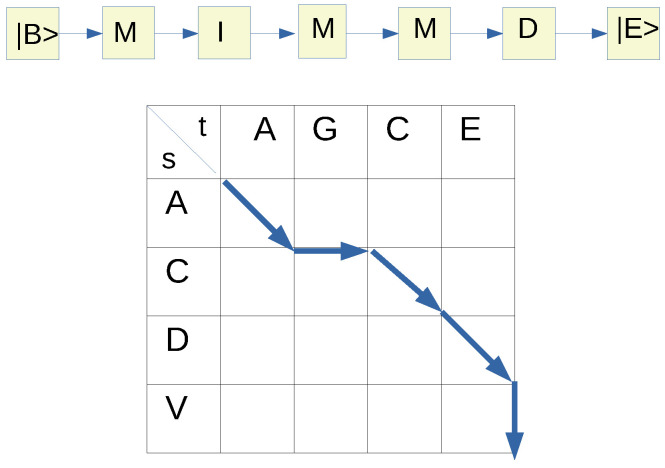
An example of simple pairwise alignment between a target sequence (*t*) and a structure sequence (*s*). Note that |B> denotes the BEGIN state and |E> denotes the END state.

Here, we present the formalism of conditional random fields as applied to pairwise sequence–structure alignment of proteins [19]. For a given pair of protein sequences *s* (which we denote, in this work, as the sequence for the known *structure*) and *t* (which we denote as the sequence for the *target* with unknown structure), an arbitrary alignment between the structural sequence *s* and the target sequence *t* can be represented as a sequence of match (*M*), insertion (*I*) or deletion (*D*) states. If we suppose that $L_{s}$ is the sequence length of the *structure*, and that $L_{t}$ is the sequence length of the *target*, then this sequence of alignment states can also be represented as an alignment path on a rectangular lattice of dimensions $L_{s}\times L_{t}$ where a diagonal path corresponds to *M*, a horizontal one to an *I* and a vertical one to a *D*. Here we assume that the target sequence (*t*) lies along the horizontal with the sequence length $L_{t}$ and the structure sequence (*s*) along the vertical with the sequence length $L_{s}$ (see Figure 1).

Now let us denote the sequence of alignment states (with alignment length *L*) as $A\equiv (a_{0},a_{1},a_{2},\cdots ,a_{L},a_{L+1})$ where $a_{i}\in \left\{M,I,D\right\}$ with i=1,⋯, *L* in addition to $a_{0}$ which indicates the BEGIN state (|B>) as well as $a_{L+1}$ denoting the END state (|E>). In the formalism of conditional random fields, a probabilistic model for the pairwise alignment is constructed where the probability P(a|s,t) of each of these alignments can be written as,

(1) $$P\left(a|s,t\right)=\exp\left[\sum_{i=1}^{L+1}F\left(a_{i-1},a_{i}|s,t\right)\right]/Z\left(s,t\right)$$

where the function $F(a_{i-1},a_{i}|s,t)$ represents the log-likelihood of the transition from the alignment state $a_{i-1}$ at i−1 to the next state $a_{i}$ at the alignment position *i* and Z(s,t) is the normalization factor with $Z\left(s,t\right)\equiv \sum_{a}\exp\left[\sum_{i=1}^{L+1}F\left(a_{i-1},a_{i}|s,t\right)\right]$, which is also called the partition function. The function $F(a_{i-1},a_{i}|s,t)$ corresponds to the alignment score at alignment position *i* in conventional pairwise sequence alignment. Here, however, it depends not just on the present alignment state $a_{i}$ but also on the previous alignment state $a_{i-1}$ through the local structural or sequence features of residues located around the alignment position i−1 and *i*. This makes it possible to naturally incorporate position dependence or environmental dependence in match scores as well as gap penalties. Important features include similarities of sequence profiles between the sequence and structure, similarities of secondary structures and solvent accessibilities. Originally, conditional random field formalism was based on the function *F* with linear combination of various scores [18]. J. Xu et al. proposed a method based on nonlinear scoring functions for *F* such as neural networks or boosted regression trees [19,20]. These nonlinear scoring functions can take nontrivial correlations between different features into account. The optimal choice of the functions *F* is obtained through training on a set of reference alignments such that the average probability of these reference alignments get maximal values.

**Figure 2:**
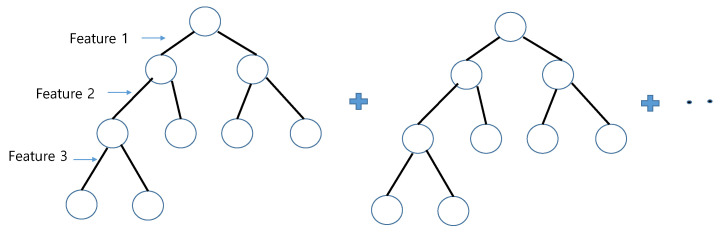
A schematic diagram of a boosted regression tree.

In our alignment, *F* consists of a sum of successive scoring functions as follows,

(2) $$F\left(a_{i-1},a_{i}|s,t\right)=T_{0}+\mu_{1}T_{1}+\mu_{2}T_{2}+\cdots$$

where $T_{0}$ is a base alignment model of scoring function and $T_{i}$’s (for i≥1) are successive nonlinear scoring functions to be determined by optimization of the probabilities of occurrences of some reference alignment set. The constants $\mu_{1}$, $\mu_{2}$, ⋯ are weight parameters that represent the learning rate of the training. Suppose that $P(a_{i-1},a_{i}|s,t)$ refers to the net probability that the specific transition from the alignment state $a_{i-1}$ at position i−1 to the next state $a_{i}$ at position *i* occur (which is also called the posterior probability). Then it is straightforward to show that, for any alignment model defined by $F(a_{i-1},a_{i}|s,t)$

(3) $$\frac{\delta \ln P}{\delta F(a_{i-1},a_{i}|s,t)}=\delta \left(a_{i-1},a_{i}\in A\right)-P\left(a_{i-1},a_{i}|s,t\right),$$

where $\delta (a_{i-1},a_{i}\in A)$ is equal to 1 if $a_{i-1}$ at position i−1 and the next state $a_{i}$ at position *i* actually pass along a reference alignment *A* and if not, it is equal to zero. A simple way to understand this relation is that, when the alignment model defined by $F(a_{i-1},a_{i}|s,t)$ is optimal (i.e., at maximum), then the right hand side of Equation (3) should be zero, in other words, $P(a_{i-1},a_{i}|s,t)$ should be equal to 1 (maximal probability) for the states on the alignment path (δ=1), and equal to zero for the states not on the alignment path (δ=0). Here, $P(a_{i-1},a_{i}|s,t)$ is obtained by summing over all the alignments of *s* and *t* with the restriction that a specific transition $a_{i-1}$ to $a_{i}$ should occur at specific position for the pair of sequences. This can be easily computed by Forward and Backward algorithm [30]. Now, we can see that the successive scoring functions $T_{k}$, k=1, 2, ⋯ can be constructed by any machine learning methods for the functional gradient of the lnP (for the alignment in question to occur) with respect to *F*, which can be easily sampled from training alignments using the right-hand-side of Equation (3). Here in this work, each $T_{k}$ is implemented as a boosted regression tree consisting of six decision trees with depth five, which is known to be fast and efficient (Figure 2).

In previous works by Xu et al. [19], the beginning alignment model $T_{0}$ for *F* was chosen as the trivial alignment model with $F_{0}\left(a_{i-1},a_{i}\right)=0$ for all possible transitions at all positions, which roughly corresponds to a random alignment model where all possible pairs of residues have equal probability of alignment as well as equal probabilities for all gaps. Here in our work, instead of beginning with the random alignment model, we chose to begin with some reasonable alignment method which is already available and build our full alignment model by adding nonlinear scorning functions within the framework of conditional random fields. Here, we chose a scoring scheme adapted from HHalign [16] as the base scoring scheme.

HHalign is a pairwise alignment method based on comparison of HMM profiles of protein sequences [16]. In order to construct a pairwise comparison of two HMM’s, HHalign introduces five pair-alignment states which are MM, MI, IM, GD and DG where *M* denotes a Match state in the HMM of a specific residue position for the structure sequence or the target sequence, *I* an Insertion state, *D* a deletion state, and finally *G* denoting a Gap state. For a given alignment between two HMM’s, the alignment score of HHalign consists of the HMM-HMM profile match score, transition probability score and secondary structure score as follows.

(4) $$T_{0}=\sum_{i}\left(S_{aa}\left(i\right)+S_{tr}\left(i\right)+S_{2}\left(i\right)\right)$$

where $S_{aa}\left(i\right)$ denotes the similarity score between the HMM profiles of the two columns at the alignment position *i*, $S_{tr}$ represents the propensity of allowed transitions at the alignment position *i* that are transitions between a pair state and itself and between pair state MM and pair states MI, IM, DG or GD. The last term $S_{2}\left(i\right)$ represents the similarity score between the secondary structure of the template (structure) residue and the predicted secondary structure of the target residue. Usually, for the template structure, the secondary structure information from DSSP [31] is used, while, for the target residue, secondary structure prediction from PSIPRED [32].

Accommodation of the HHalign-type scoring into our CRF alignment model with additional nonlinear scoring function may be implemented in two different ways that are called in this work as the three-state scheme or the five-state scheme. In the three-state scheme of CRF alignment model, we reduce the five states MM, MI, IM, DG, and GD of HHalign to the usual three states *M*, *I*, *D* via the reduction mapping of (MM→Match(M), MI→Insertion(I), IM→Deletion(D), DG→Insertion(I), GD→Deletion(D)). Note that MI and DG are both reduced to the same alignment state of *I*, while IM and GD to *D*. For a given alignment path in this three-state scheme, the zeroth order scoring is obtained by reduction of the HHalign scores such that for an Insertion (*I*) or a Deletion (*D*) state, the larger score of the two corresponding states in the original five-state model from HHalign is chosen.

It is also possible to construct a CRF alignment model with a five-state scheme (i.e., without reduction to the three states) incorporating the full HHalign scoring and additional nonlinear scoring functions. Moreover, in this scheme of five state, for the purpose of training from the reference alignments, it is necessary to assign an appropriate five-state label for each of the alignment positions between the target and the template. Since the reference alignments are built by some structure alignments without relation to the HMM profiles of the targets or templates, it is not straightforward to assign five state labels to the alignment states due to the ambiguity between DG vs. MI as well as GD vs. IM. One possible solution to this problem is to choose the unique assignment along the reference alignment path for which the HHalign score becomes maximum. We implemented and tested both the three-state model and the five-state model.

Once the base alignment model for the three-state scheme is fixed, additional nonlinear scoring functions ($T_{1}$, $T_{2}$, ⋯) can be constructed via training on a set of reference alignments as follows. One can evaluate the right-hand side of the Equation (3) based on the base alignment model $T_{0}$ to get the functional derivative $\delta \ln P/\delta F(a_{i-1},a_{i}){|}_{F=T_{0}}$ for any pairwise alignment. Now, we sample transitions from the set of reference training alignments. Both positive samples (i.e., those transitions appearing in the training alignments) as well as negative samples (i.e., those transitions that are not appearing in the training alignments) are taken. For these samples, one can compute the right-hand side of the Equation (3) $1-\langle P_{a_{i-1},a_{i}}\rangle$. These target values together with the relevant input features can now be used to train the first additional scoring function $T_{1}$ i.e., first correction to the alignment model, where any machine learning methods can be employed. The constant factor $\mu_{1}$ can be chosen to control the degree of greediness of the training.

For training these gradients, we used so-called gradient boosted regression trees [24]. In addition, the partition function Z(s,t) can be calculated using the standard Forward-Backward algorithm for the given alignment model. Now, when this training is completed for $T_{1}$, we are now equipped with a first order corrected alignment model, which can again be used for training the next order regression trees $T_{2}$ for further correction, using new samples evaluated at $T_{0}+\mu_{1}\cdot T_{1}$. The constants $\mu_{i}$ (*i* = 1, 2, 3, ⋯) are weight parameters that can be adjusted to control the degree of convergence in the training, where we chose $\mu_{i}=0.2$ for all *i* in this work. For each of the three (for the three-state scheme) or five (for the five-state scheme) alignment states, there corresponds a boosted regression tree. Input features for the regression trees include position-dependent structural features such as secondary structures and solvent accessibilities for the known protein structure. In addition, for the sequence with unknown 3D structure, predicted secondary structures and solvent accessibilities are employed instead. In all, there are six features for the Match state and there are another six features for the Gap state. For further details on the input features to the boosted regression trees, refer to Appendix A.

In the CRF alignment model, two kinds of alignments are possible. One is the so-called Viterbi alignment algorithm which selects the one highest scoring alignment (i.e., highest probability). The other method of alignment is the so-called MAP (MAximum Posterior Probability) alignment which first calculates the net probability $P(s_{i},t_{j})$ for a specific pair of residues $s_{i}$ (of the template structure) and $t_{j}$ (of the target) may align in all possible alignments, and then find, through a standard dynamic programming, the alignment that optimizes the sum of these values without gap costs,

(5) $$S\left(a\right)\equiv \sum_{a}P\left(s_{i},t_{j}\right)$$

where the sum is performed over all pair matches. This is also called as Maximum Accuracy Alignment (MAC). MAP alignment tends to produce more true matches compared with the Viterbi alignment (see the section on Results).

In contrast to global alignment (where the alignment begins at the first residues of the target and the template), local alignments can be generated by allowing the alignment to begin (and end) at any position of the target and the template without scoring for the end gaps. In the case of MAP alignment, this can be conveniently implemented by introducing a threshold value $m_{th}$ for the match which can range from 0 to 1 and subtract $m_{th}$ from $P(s_{i},t_{j})$ for all matches as follows,

(6) $$S_{local}\left(a\right)\equiv \sum_{a}\left(P\left(s_{i},t_{j}\right)-m_{th}\right)$$

where, in the dynamic programming, the alignment can begin at any position of the target and template with no costs for end gaps. As for internal gaps, additional penalties of $-0.5*m_{th}$ are added to avoid unnatural internal gaps being produced. A special case of alignment mode which is called *glocal* (*glocal + local*) alignment is commonly adopted, where the alignment can begin (and end) at internal positions of either the target or the template but not *both*. If we suppose that the sequence of the structure template runs along vertical axis on the left boundary of a rectangular lattice, with that of the target running along horizontal axis on the top boundary, this corresponds (in terms of the alignment path) to beginning the alignment on the upper or left boundaries of the rectangle and ending on the opposite sides (bottom or right boundaries) in the dynamic programming.

## 3. Results

As for the reference alignment set for training our sequence–structure alignment method, we chose the SABmark (version 1.65) benchmark set [25] which was designed to assess protein sequence alignment algorithms, especially for the case of remote homologous pairs of proteins. SABmark consists of two sets of pairwise and multiple alignment sets with high-resolution X-ray structures derived from the SCOP classification. These sets, Twilight Zone and Superfamilies, are known to cover the entire known fold space with sequences very low to low and low to intermediate similarity, respectively.

The Twilight Zone set consists of 209 sequence groups that each represent a SCOP fold. Sequence similarity is very low with the sequence identities ranging between 0% and 25% and also with the structures being distantly similar. SABmark homepage states that “This set therefore, represents the worst case scenario for sequence alignment, which unfortunately is also the most frequent one, as most related sequences share less than 25% identity” [25]. On the other hand, the Superfamilies set consists of 425 groups, each of which representing a SCOP superfamily. The sequence pairs share at most 50% identity. Even though this set in general consists of less difficult pairs (than the Twilight Zone) they still represent challenging problems for sequence alignments.

We chose three sets of reference alignment, each consisting of 200 pairwise alignments from the Superfamilies set and from the Twilight Zone. These three sets are labeled as NG200, NF200 and TW200, respectively. These are chosen in such a way that the pairs are evenly distributed among different groups of families so that as many groups of families as possible are covered. Among these three sets, two of them (NG200 and NF200 set) are from the Superfamilies set with average sequence identities of 24.2%, 21.2%, respectively. The remaining set of TW200 is derived from the Twilight zone set with average sequence identity of 13.8%. By training our sequence–structure alignment methods on these sets with different levels of sequence similarity, we may be able to compare the modeling capabilities of the resulting alignment methods and choose the most efficient one among those results.

HMM files were generated by using hhmake tools of HMM hhsuite [16,23]. In our training on the three sets (NG200, NF200 and TW200), we employed the three-state scheme. That is, at each step of the training, e.g., $T_{i}$ with i=1, 2, ⋯, there are three different boosted regression trees, one for each of the three states at the current position: match (*M*), insertion (*I*) and deletion (*D*) state, respectively (i.e., the three-state scheme).

For each pairwise alignment of the reference training set, we take each of the alignment states along the reference alignment path as a positive sample (for training the scoring function). If it is a match state (*M*), we add the set of corresponding features together with the target label value $1-P(a_{i-1},a_{i})$ in Equation (3) to the sample set for the boosted regression tree for the match state. Similarly for insertion (*I*) or deletion (*D*) states along the reference alignment path, we add the corresponding features and the label value to the boosted regression trees for *I* and *D* respectively. Now, we have to also collect negative samples, that is, those transitions that do not appear on the reference alignments. Suppose that the sequence length of the structure *s* and the target *t* are $L_{s}$ and $L_{t}$ respectively. If we let the alignment length to be $L_{a}$, then we have $L_{a}\leq L_{s}+L_{t}$. Then, we can see that (e.g., for three state alignment model) there are about $≃3(L_{s}\cdot L_{t}-L_{a})$ negative samples which is usually much larger than the alignment length $L_{a}$. We randomly selected some integer ($N_{f}$) times the alignment length $L_{a}$ for the size of the negative samples, where we took $N_{f}=16$. That is, we took $16\cdot L_{a}$ transitions that are not on the alignment path. These transitions were distributed evenly among the three alignment states *M*, *I* and *D*. We tried other values for $N_{f}$, but the present value was found to be most effective in terms of the training accuracy and training time. This resulted in around $2\times 10^{5}$ samples for each of the three states. At each step of the training, the boosted regression trees consist of six regression trees with each tree having a depth of five. As for the choice of the parameters $\mu_{k}$, as mentioned above, we simply set $\mu_{k}=0.2$ for all steps *k*. Change of this parameter did not show much difference in the performance of the resulting alignment model.

For each of the above three sets (NG200, NF200 and TW200), in order to perform training of our alignment model and then perform validation test in terms of alignment accuracies, we divided the set into four subsets of 50 pairs each, and then carried out a four-fold training and test with 150 pairs for training and the remaining 50 pairs for test in turn. Alignment accuracies are measured as the ratio of the number of aligned pairs out of the true aligned pairs in the reference structure alignment of the sequence pairs of the SABmark benchmark. For training and test on these sets presented, we employed a three-state scheme.

**Figure 3:**
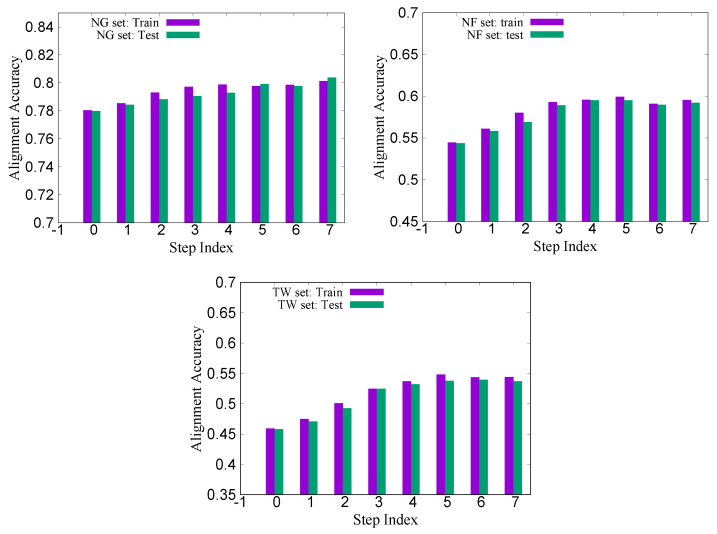
Training and test accuracies for the Viterbi alignment on the three sets NG (**top left**), NF (**top right**) and TW (**bottom**) of 200 reference alignments from SABmark. Note that we are using the three-state scheme here.

**Figure 4:**
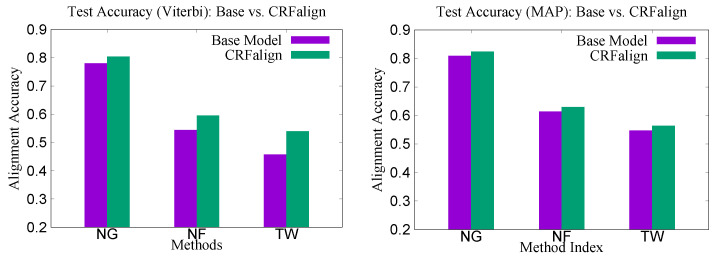
Training and test accuracies at Maximum for (**left**) the Viterbi alignment as well as (**right**) the MAP alignment on the three sets (NG, NF and TW) of 200 reference alignments with the three-state scheme.

Figure 3 shows the training and test accuracies (with Viterbi scoring) for the three sets (NG200, NF200 and TW200) as the training step increases. We see that on all three cases, the training and the test accuracies on average improve up to a certain regression steps (about five to seven, depending on the sets), then after that, the average accuracies tend to fluctuate somewhat. Figure 4 shows the relative improvements of the CRFalign test accuracies over the base (zeroth order) model for the three sets. We can clearly see that, for the hard alignment set of NF200 and TW200 as compared with the easier set of NG200, more improvement is achieved especially in terms of the Viterbi alignment accuracies.

In order to assess our alignment method in terms of protein structure modeling for proteins in the SABmark set, we chose the whole 200 pairs of the NF200 set to train our alignment model and then performed sequence–structure alignment with the trained model together with structure modeling on two independent test sets using the Modeller program based on the alignment. One of the two test sets called NG64 consists of 64 pairs chosen from the Superfamilies set of the SABmark. On the other hand, the second test sets called TW55 consists of 55 pairs chosen from the Twilight Zone set of the SABmark, representing more difficult alignment situations. Both sets consist of pairs of sequences that are less than 30% sequence identity against those of the training set NF200 with the average of the sequence identity against the training set being 16.6% (NG64) and 16.3% (TW55), respectively.

**Figure 5:**
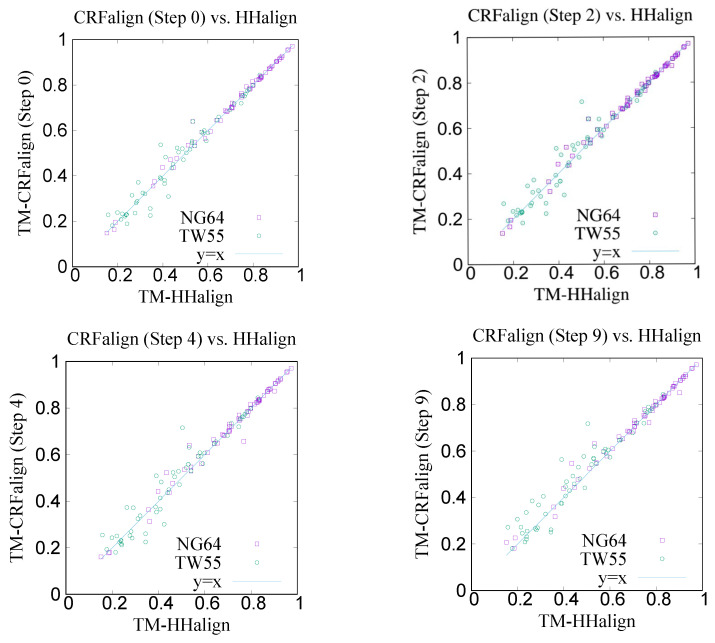
TM scores of structure models of NG64 set and TW55 set obtained by running Modeller on the CRFalign alignments (three-state scheme) at various train steps (**top left**: steps 0, **top right**: step 2, **bottom left**: step 4 and **bottom right**: 9) in comparison with the results of HHalign.

Figure 5 shows the modeling results on NG64 and TW55 test sets comparing the CRFalign method with HHalign, where we see that some improvements were made by CRFalign over HHalign results. Table 1 shows the average TM score for the modeling results where again we find that for the hard set of TW55 the improvement was bigger. The average TM score of NG64 set by CRFalign was 71.96%, while that for HHalign was 71.39%. On the other hand, the average TM score of TW55 set by CRFalign was 48.83%, while that for HHalign was 46.32%.

**Figure 6:**
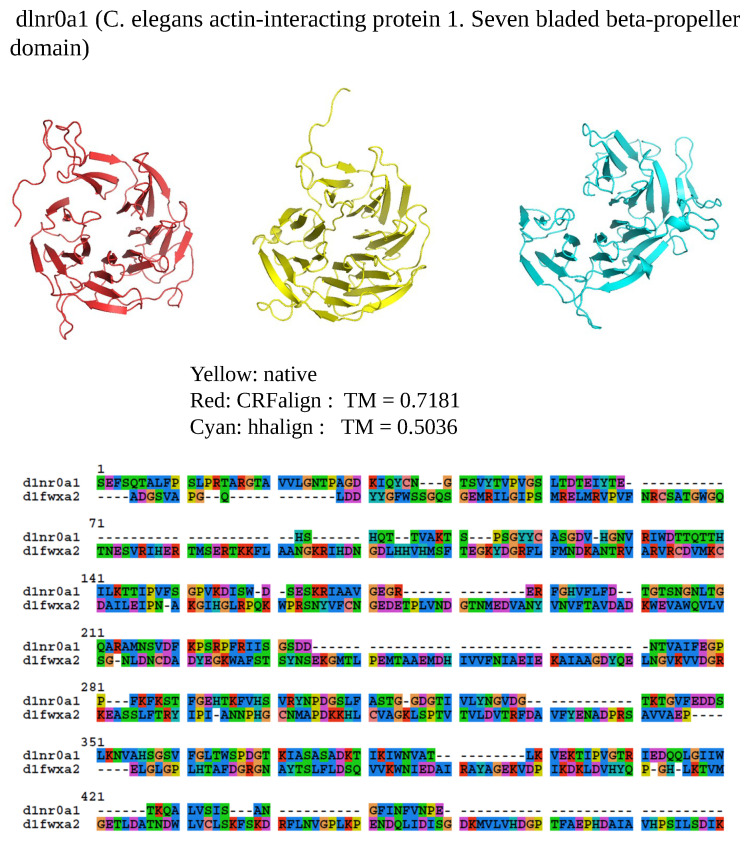
Structure models of d1nr0a1 (C.elegans actin-interacting protein1 Seven-bladed beta-propeller domain) based on d1nr0a1-d1fwxa2 alignment (Reference TM = 0.75381, ID = 9.4%) from CRFalign (**top left**, red) with TM-CRFalign = 0.7181, and HHalign (**top right**, cyan) with TM-HHalign = 0.5036; at the to center (yellow) is the native structure. The **bottom** figure shows the CRFalign pairwise alignment where a few large gaps are recognized for proper alignment.

Figure 6 shows one example where CRFalign result was fed into Modeller with the resulting model exhibiting significant improvement over that of HHalign. Shown is the 3D structure of the chain A of d1nr0a1 (which is the seven-bladed beta propeller domain of C. elegans actin-interacting protein 1) with the template d1fwxa2 (d1nr0a1-d1fwxa2, ${\mathrm{TM}}_{ref}=75.38\%$, ID=9.4%). Note that the sequence ID to the template sequence is only 9.4% but still CRFalign in combination with Modeller could produce a structure of TM score with TM≃71.8% which is close to the optimal TM score limit of ≃75.38%. In contrast HHalign could not properly close the propeller shaped domain with relatively poor value of the TM score of TM-HHalign =50.36%. In the CRFalign alignment between the template and our target sequence (not shown), we could see that there are a few large gaps in the alignment which would be difficult to correctly align without the help of structure based features and nonlinear scoring model for the alignment.

**Figure 7:**
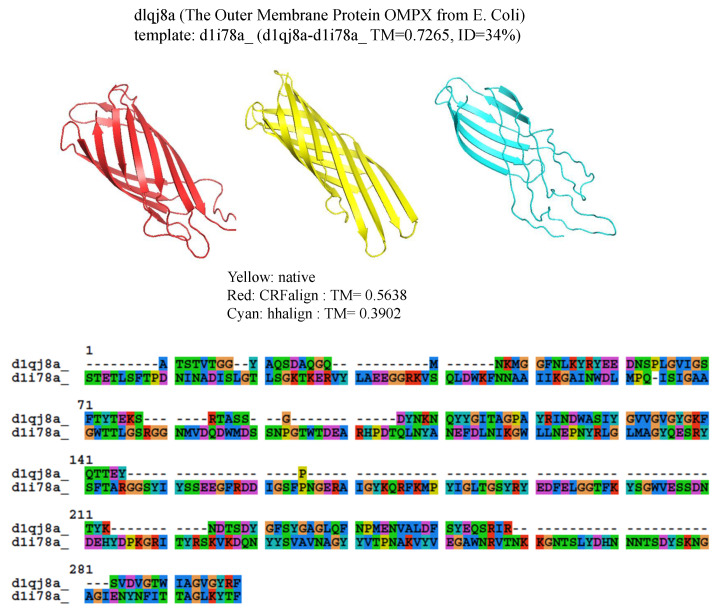
Structure models of d1qj8a (The Outer Membrane Protein OMPX from E. Coli) based on d1qj8a-d1i78a_ alignment ( Reference TM = 0.7265, ID = 3.4%) from CRFalign (**top left**, red) with TM-CRFalign = 0.5638, and HHalign (**top right**, cyan) with TM-HHalign = 0.3902; at the top center (yellow) is the native structure. At the **bottom** is shown the CRFalign alignment where also a large gap is recognized.

Another example is shown in Figure 7 where β protein (The Outer Membrane Protein OMPX from E. Coli 1qj8a) is illustrated based on the alignment (d1qj8a-d1i78a ${\mathrm{TM}}_{ref}=72.65\%$, ID=3.4%) which could roughly produce the correct fold pattern with TM-CRFalign = 56.38% as compared with HHalign, which failed in producing the correct β patterns on one side with TM-HHalign = 39.02. In this case, the sequence identity is even lower with ID=3.4%. Here also, the CRFalign alignment to the template (not shown) shows regions of big gaps.

**Figure 8:**
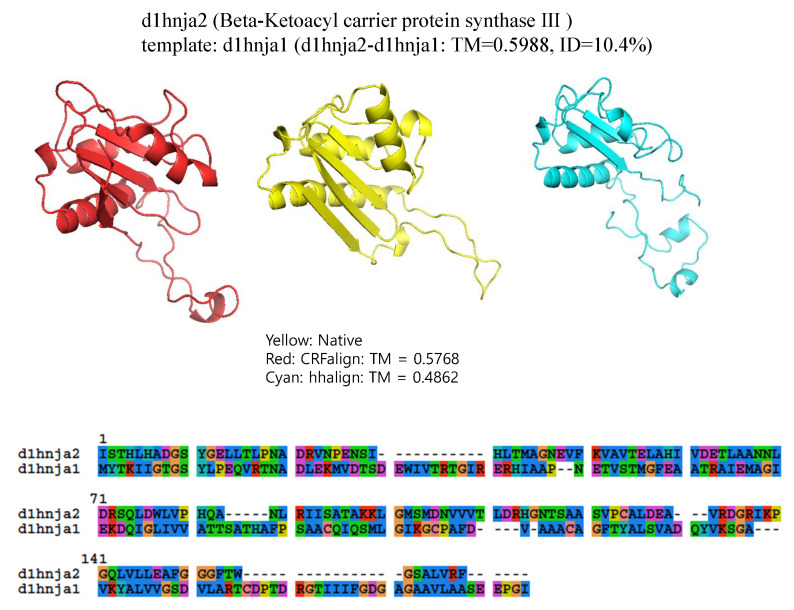
Structure models of d1hnja2 (Beta-Ketoacyl-acyl carrier protein synthase III) based on d1hnja2-d1hnja1 alignment (Reference TM = 0.5988, ID = 10.4%) from CRFalign (**top left**, red) with TM-CRFalign = 0.5768, and HHalign (**top right**, cyan) with TM-HHalign = 0.4862; at the top center (yellow) is the native structure. The **bottom** figure shows the CRFalign alignment.

The final example is shown in Figure 8, which shows the structure of d1hnja2 (Beta-Ketoacyl-acyl carrier protein synthase III) with the alignment d1hnja2-d1hnja1 (${\mathrm{TM}}_{ref}=59.88\%$, ID = 10.4%). Here, CRFalign could produce TM-CRFaligna = 57.68% which is quite close to the ideal value of ${\mathrm{TM}}_{ref}=59.88\%$. In contrast, HHalign could produce the model with TM-HHalign = 48.62% only, failing to reproduce much of the secondary structural elements.

For building working alignment models (targeted for blind structure prediction such as CASP), we trained several hundred different three-state alignment models (i.e., accumulating different sets of boosted regression trees) on the three sets (NG200, NF200 and TW200) using the whole 200 pairwise alignments for each of the three sets. In order to choose optimal alignment models, we need to test these for their modeling capabilities. For this, we tested these on CASP10 targets with appropriate templates by performing alignment and modeling. We chose 58 single-domain targets from CASP10, for which there exist templates. Among these, for 50 of them, we could choose two templates. Hence, in all, we have 108 pairs to align and model.

**Figure 9:**
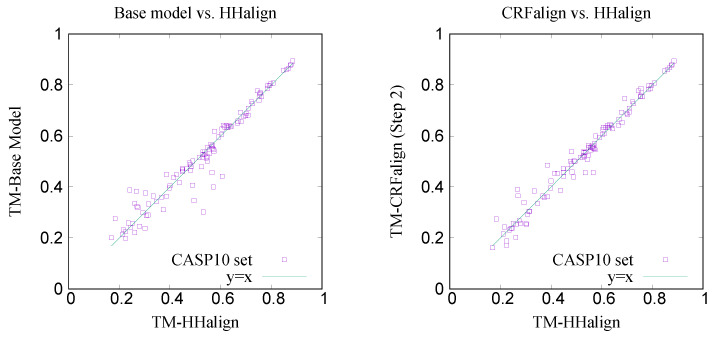
Comparison of TM scores for CASP10 targets by Modeller modeling based on (**left**) base alignment (average TM score =0.5246) vs. HHalign alignment (average TM score =0.5286) and (**right**) CRFalign alignment (average TM score =0.5321) vs. HHalign.

Figure 9 shows the comparison of the TM scores for the modeled structures with the base alignment model vs. HHalign where we see that the average TM score with the base alignment model (${\mathrm{TM}}_{base}≃0.5246$) is lower than that of the HHalign model (${\mathrm{TM}}_{hha}≃0.5286$). However, on the right side, shown is the comparison between the CRFalign (three-state) result and HHalign, where we see various targets for which the base model gave relatively poor result are now showing some improvement with the resulting average TM score of TM-CRFalign ≃0.5321. This CRFalign alignment with the three-state scheme was applied successfully to CASP11 [27] and CASP12 [33].

**Figure 10:**
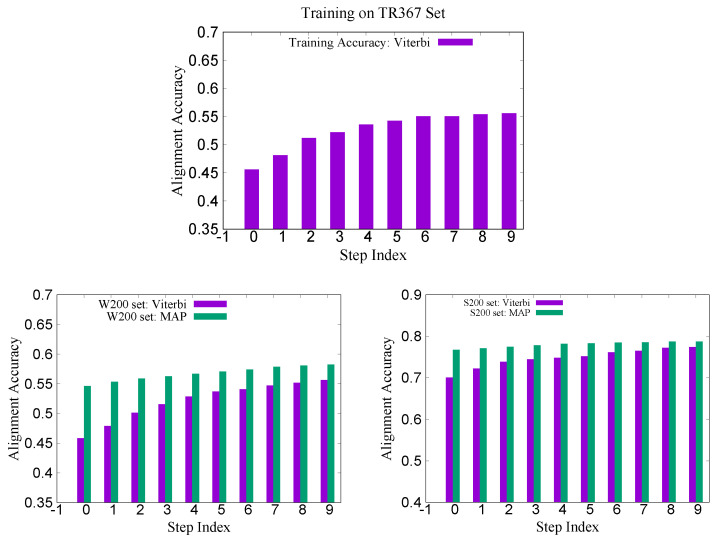
(**Top**) Training alignment accuracy on TR367 set with the five-state model training. (**Bottom left**) Test alignment accuracies on W200 set with the same five-state model; both Viterbi alignment and MAP alignment accuracies are shown. (**Bottom right**) Test alignment accuracies on S200 set with the same five-state model (below).

Recently, we constructed a larger training set called TR367 from SABmark for CRFalign with the five-state alignment model. The training set TR367 consists of 367 pairs of proteins from SABmark superfamily (299 pairs) and twilight zone set (68 pairs). These were carefully selected more or less uniformly among different folds and families. In order to assess the pair-wise alignments of the five-state alignment model, we prepared two test sets W200 and S200 from SABmark benchmark set. The W200 set consists of 200 pairs chosen from the twilight zone subset of SABmark set, while those of S200 are 200 pairs from the superfamily set, where all the sequences in the test set have sequence identities less than 20% against those sequences in the training set (TR367). Therefore, the pairs in W200 set should be considered, in general, harder (i.e., remote homologues) than those of S200 set. Figure 10 shows the training and test accuracy of alignments for the TR367 training set and the W200 test set as well as S200 test set. We can see here also that the average alignment accuracy in the W200 set shows larger relative improvement than that in the case of S200 set (Table 2).

**Figure 11:**
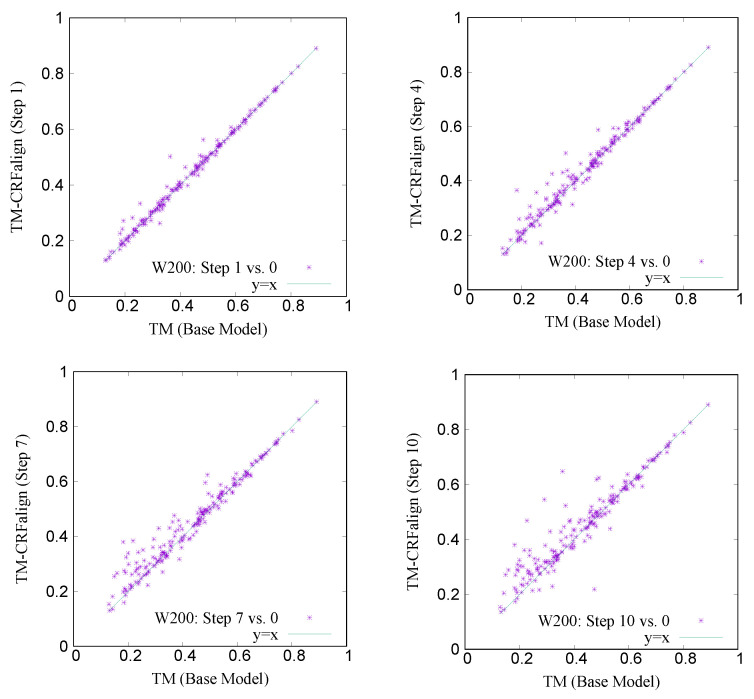
TM scores of structure models on the W200 test set with CRFalign (the five-state model) at steps 1 (**top left**), 4 (**top right**), 7 (**bottom left**), 10 (**bottom right**) vs. the Base model (step 0), respectively.

Table 3 shows the modeling accuracies on the two test sets W200 and S200. We can recognize larger relative improvement in the TM score in the case of W200 set compared with that of S200 set which is consistent with the alignment accuracies shown above. Figure 11 shows a comparison of the TM scores of individual targets for W200 set based on CRFalign (at different steps of 1, 4, 7 and 10) vs. the Base model. Here also, one can recognize significant relative improvement in the targets of low TM score region.

**Figure 12:**
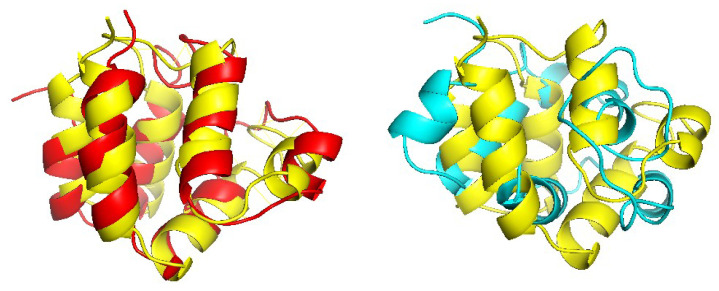
(**Left**) Structure model of d1a1w__(FADD death-effector domain) based on d1a1w__-d1dgna_ CRFalign alignment (red) overlapped with the experimental structure of d1a1w__ (yellow) (with TM score = 0.6477, rmsd=2.318 ) and (**right**) that based on base model alignment (cyan) overlapped with the experimental structure (yellow) (with TM score = 0.3575, rmsd=12.111).

**Figure 13:**
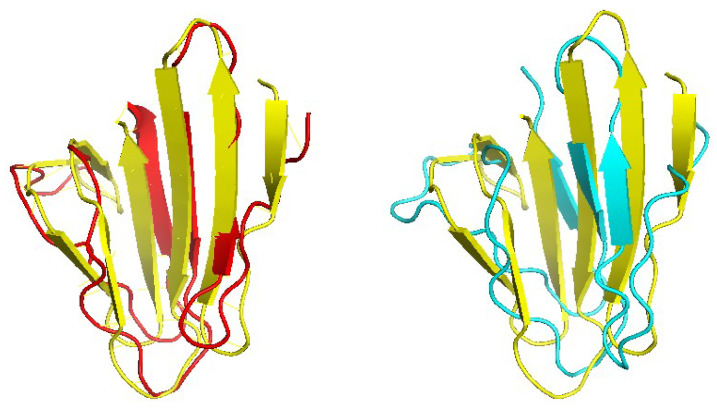
(**Left**) Structure model of d1gjwa1 (Thermotoga maritima maltosyltransferase) based on CRFalign alignment (red) of d1gjwa1-d1ktba1 overlapped with the experimental structure of d1gjwa1 (yellow) (with TM score = 0.6243, rmsd=2.781 ) and (**right**) that based on the base model alignment (cyan) overlapped with the experimental structure (yellow) (with TM score = 0.4907, rmsd=4.616 ).

**Figure 14:**
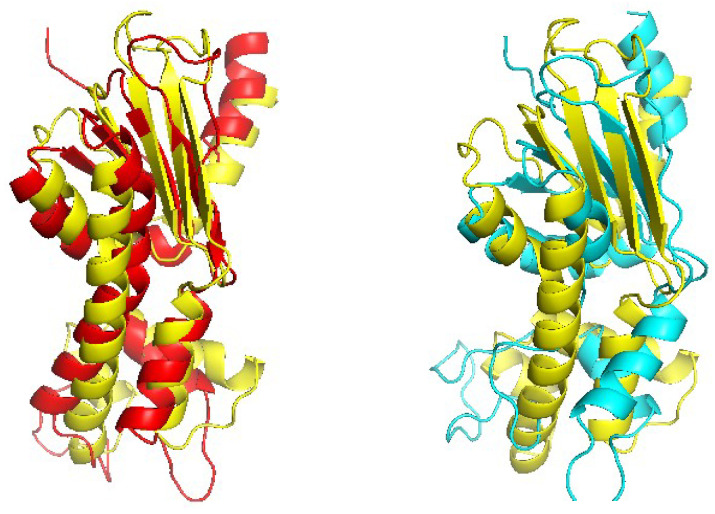
(**Left**) Structure model of d1mwma2 (ParM from plasmid R1 ADP form) based on CRFalign alignment (red) of d1mwma2-d1nbwa3 overlapped with the experimental structure of d1mwma2 (yellow) (with TM score = 0.6182, rmsd=3.749) and (**right**) that based on Base alignment (cyan) overlapped with the experimental structure (yellow) (with TM score = 0.4829, rmsd=7.159 ).

Figure 12, Figure 13 and Figure 14 show examples of significantly improved model structures from the W200 set using CRFalign (and Modeller) with the five-state model as compared with those from the base model. The first example domain is d1a1w__(FADD death-effector domain) which consists of mostly alpha-helices. The structure model produced from CRFalign with its template (d1dgna_) exhibits TM score of 0.6477 and rmsd=2.318 with a sizable improvement over that from the Base alignment with TM score = 0.3575 and rmsd=12.111 (Figure 12). The next example is d1gjwa1 (Thermotoga maritima maltosyltransferase) which consists of mostly beta sheets. In this case also, we observe that the CRFalign (of d1gjwa1-d1ktba1) leads to a model structure with TM score = 0.6243 and rmsd=2.781 which is a significant improvement over that based on the base model alignment with TM score = 0.4907 and rmsd=4.616 (Figure 13). The final example is d1mwma2 (ParM from plasmid R1 ADP form), which consists of alpha and beta structures. The CRFalign alignment (of d1mwma2-d1nbwa3) and Modeller produce a model with TM score = 0.6182 and rmsd=3.749 in comparison with that based on the Base alignment with TM score = 0.4829, rmsd=7.159 (Figure 14).

We also tested the performance of CRFalign (five-state scheme) on hard targets of CASP14 [29], where the targets are selected from the 15 protein domains listed as FM (Free Modeling) or FM/TBM (of CASP14). These target domains are T1026-D1, T1030-D1, T1032-D1, T1033-D1, T1038-D2, T1039-D1, T1046s1-D1, T1046s2-D1, T1056-D1, T1067-D1, T1074-D1, T1079-D1, T1080-D1, T1082-D1 and T1099-D1. The structural homologues of these targets (obtained by using LGA structure alignment [34]) can be retrieved from CASP14 homepage (https://predictioncenter.org/download_area/CASP14/templates/LGA/, last accessed on 1 February 2022). For each of these domain targets, 1–6 homologues are available for templates. We ended up with 51 pairs of target and templates involving 15 CASP14 domains for sequence–structure alignment. We compared the TM scores of the structure models obtained via Modeller based on the pairwise alignments using CRFalign and HHalign, respectively. Table 4 shows the average TM score of the structure models from CRFalign at each step number (with both the five-state and three-state models). This can be compared with the TM score result for HHalign TM-HHalign = 0.3905 which is close to the result of base model (in three-state alignment model). For reference and comparion, we tested (baseline) pairwise alignments using BLOSUM62 scores [35] and also recent MRFalign alignment on the above set. These alignments produced (through Modeller) 3D models with average TM scores of TM-Blosum62 = 0.3077 and TM-MRFalign = 0.3693 Table 5. We see that the highest average TM score for CRFalign (five-state model) is around TM = 0.4294 (at step 9) which improves upon HHalign by nearly 3.9% point, and upon MRFalign by about 6.0% respectively as shown in Table 5. The maximal possible average TM score using TM-align (=0.5973) is also shown in Table 5 which shows that there are significant gaps between the maximal TM score and CRFalign. However, it is important to note that these CASP14 targets are classified as hard targets where the corresponding templates are very hard to find from typical template searches, and that these templates are identified from structural alignments such as LGA.

**Figure 15:**
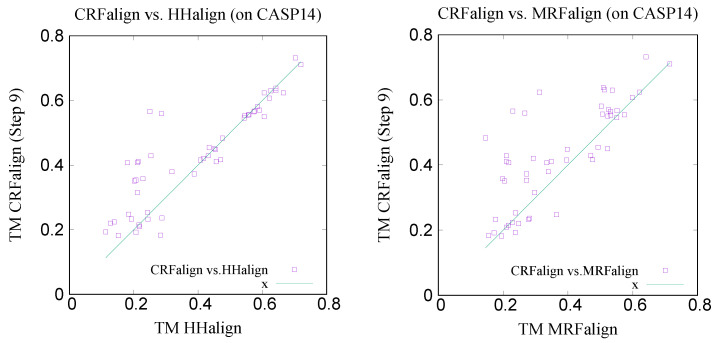
Comparison of the TM scores of structure models on hard targets of CASP14 by CRFalign (five-state model, step 9) against HHalign (**left**) and MRFalign (**right**) respectively.

**Figure 16:**
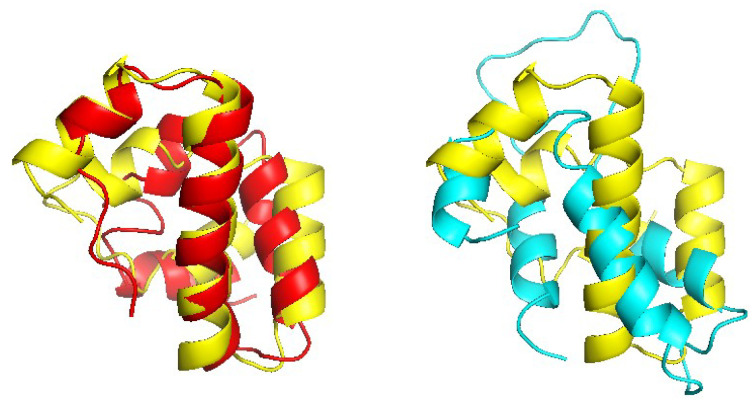
(**Left**) Structure model of the target T1082-D1 based on CRFalign alignment of T1082-D1-6h7bC (red) overlapped with the experimental structure of T1082-D1 (yellow) (with TM score = 0.5656, rmsd=3.01) and (**right**) that based on HHalign alignment (cyan) overlapped with the experimental structure (yellow) (with TM score = 0.2499, rmsd=8.83 ).

Figure 15 shows the x-y comparison plot of the TM scores of the 51 pairs by CRFalign (with the five-state scheme at step 9) against those of HHalign (left) and another comparison of CRFalign against MRFalign [21] (right). We can see that CRFalign improves the modeling accuracy significantly for some of the hard targets in comparison with both HHalign and MRFalign. Figure 16 shows an example of the modeled structures among the CASP14 hard targets which exhibit notable improvement in the modeling accuracies. This target is T1082-D1, which consists of alpha helices where CRFalign results in TM score = 0.5656 that exhibits a large improvement over TM score = 0.2499 of HHalign. We used the above set of 51 pairs of CASP14 target-templates for estimating the running speed of CRFalign alignments. With the average sequence length per target of 178 residues, the average running time of a pairwise alignment was 1.76 seconds on our single CPU of AMD EPYC 7543 (2.80 Ghz).

Sequence–structure alignment is useful in fold recognition i.e., template search. In order to test the template search capability of CRFalign alignment, we incorporated CRFalignment (with the five-state scheme) into our fold recognition framework called CRFpred [27,33]. CRFpred utilizes a set of machine learning methods such as random forest, boosted regression tree, support vector machine and linear regression, on features obtained from CRFalignment output. These features include profile scores, secondary structure scores and solvent accessibility scores. Details of the CRFpred will be presented in future publications. The structure database of 35539 proteins was built with 40% sequence identity cutoff. We randomly selected 300 targets from the database with the sequence length ranging from 100 to 500. These targets can be divided into three groups according to the levels of difficulties as measured from TM scores between the targets and best templates (excluding the targets themselves) among the database. These three sets are EASY (170 targets, TM > 80%), MEDIUM (89 targets, 60% < TM < 80%) and HARD (41 targets, TM < 60%) sets.

**Figure 17:**
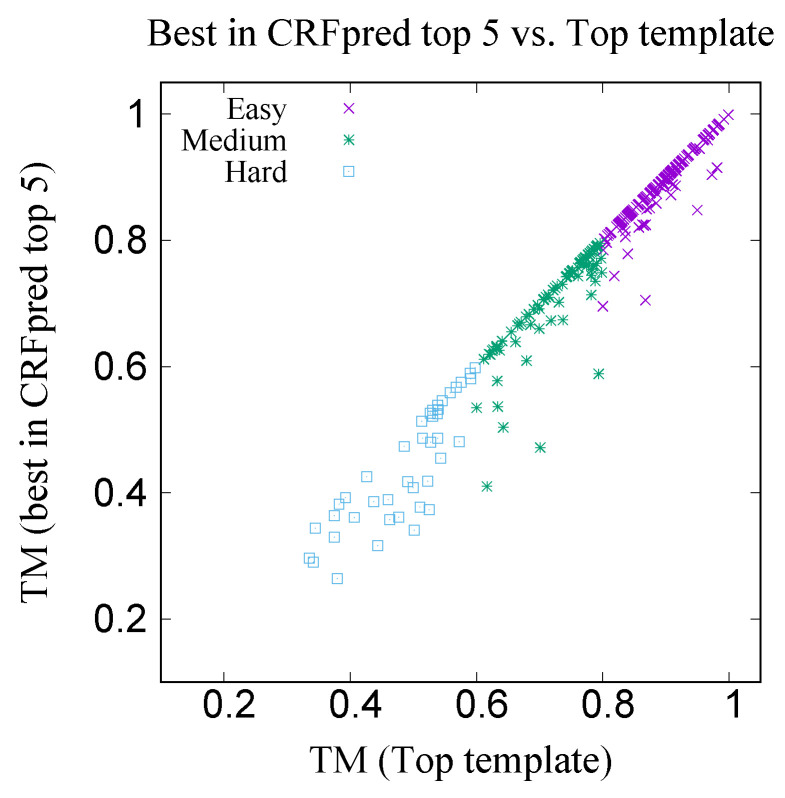
Comparison of the TM scores of 300 proteins with the best among top five predicted templates from CRFpred (through CRFalign) vs. the TM scores of the same proteins with the (true) top templates from the database.

**Figure 18:**
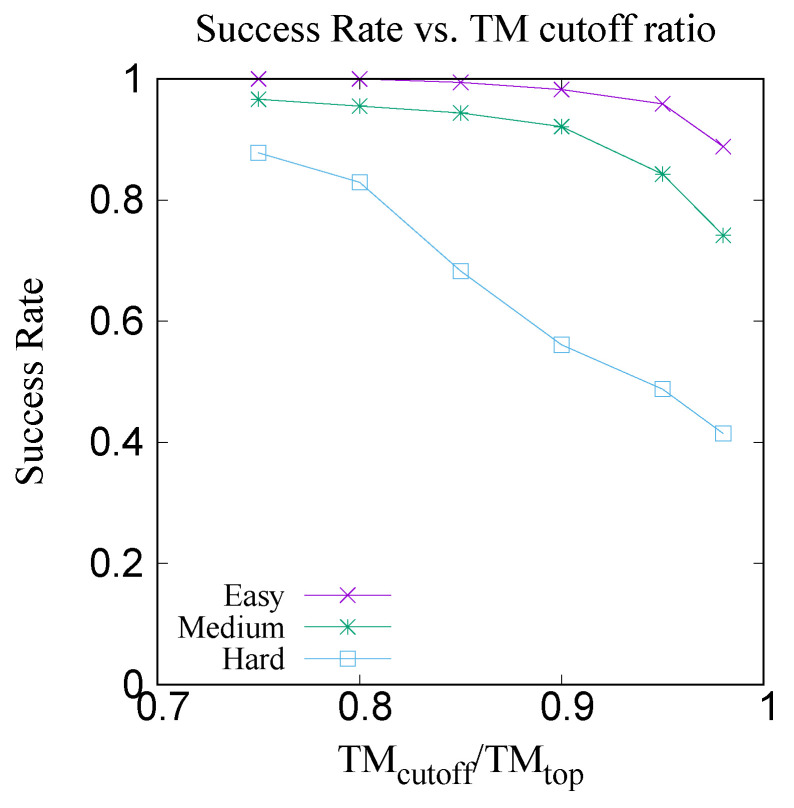
Success rate of finding (among the top five CRFpred templates) a template that is within a certain cutoff ratio of the maximal possible TM score for three subsets of Easy, Medium and Hard targets.

For each target, search is made from the database (excluding the target itself) and the top five templates are chosen from the result of CRFpred search. Table 6 shows the average TM scores of the targets with the best predicted (CRFpred) template and with the top templates respectively. Figure 17 shows the xy-plot comparison of the TM scores of 300 target proteins with the best template among the top five predicted templates by CRFpred (CRFalign) vs. the TM scores of the same proteins with the top templates from the database. In order to check how well CRFalign and CRFpred can detect a template that is close enough to the top template, we plot in Figure 18, for each of the three taregt sets, the success rate of finding a template wth a TM score that exceeds a given cutoff ratio of the TM score of the top template. We can see that, for the Easy target set, the detection rate at cutoff ratio of 95% reaches 95.9%, while for the case of the Medium target set, at the same cutoff ratio of 95%, the detection rate is down at 84.3%. On the other hand, for the case of the Hard target set, the detection rate at cutoff ratio of 95% is only 48.8%. By lowering the cutoff ratio to 85%, we can see that the detection rate increases to 68.3%. This manifests some measure of the difficulty in detecting a reasonable template for the case of the Hard target set.

## 4. Discussion

A sequence–structure alignment method CRFalign is presented that improves upon a reduced three-state or five-state scheme of HMM-HMM profile alignment model by means of conditional random fields with nonlinear scoring on the sequence and structural features implemented with boosted regression trees. CRFalign can extract and exploit complex nonlinear relationships among sequence profiles and structural features, including secondary structures, solvent accessibilities, environment-dependent properties that give rise to position-dependent and environment-dependent match scores and gap penalties. Training of the CRFalign is performed on a chosen set of reference pairwise alignments from the SABmark benchmark set, which consists of Twilight Zone set and Superfamilies set with pairs of sequences very low to low, and low to intermediate sequence similarity, respectively. We found that our alignment method produces relative improvement in terms of average alignment accuracies, especially for the alignment of remote homologous proteins. Comparison of the modeling capabilities of our alignment on independent pairs of SABmark set with those of HHalign showed that our alignment method produced better modeling results especially in the relatively hard targets. This was also confirmed in recent tests on hard targets of CASP14. CRFalign was successfully applied to the initial stages of fold recognition and as an input to the multiple sequence alignment called (MSACSA) in the CASP11 and CASP12 competition on protein structure predictions.

## Figures and Tables

**Table 1:** Modeling accuracies in TM score of the test sets NG64 and TW55 based on training with NF200.

Set	HHalign	Base Model	CRFalign (3-State Scheme)
NG64	0.7139	0.7176	0.7196
TW55	0.4632	0.4669	0.4883

**Table 2:** Alignment accuracies in the test sets W200 and S200 based on training with TR367.

Set	Base Model	CRFalign (Five-State Scheme)
W200	0.546	0.583
S200	0.767	0.787

**Table 3:** Modeling accuracies in average TM score of the test sets W200 and S200 based on the five-state training with TR367 set.

Step Number	W200	S200
0	0.4304	0.5776
1	0.4331	0.5792
2	0.4368	0.5812
3	0.4372	0.5808
4	0.4377	0.5809
5	0.4406	0.5826
6	0.4431	0.5827
7	0.4455	0.5820
8	0.4473	0.5833
9	0.4462	0.5849
10	0.4499	0.5870

**Table 4:** Modeling accuracies in average TM score of 51 pairs involving the CASP14 hard targets based on the five-state model as well as the three-state model.

Step Number	CRFalign (5-State)	CRFalign (3-State)
0	0.3938	0.3902
1	0.3983	0.3958
2	0.4009	0.4029
3	0.4027	0.4041
4	0.4082	0.4045
5	0.4107	0.4071
6	0.4156	0.4074
7	0.4201	0.4011
8	0.4253	0.4034
9	0.4294	0.4129
10	0.4237	0.4198

**Table 5:** Average TM score of models by various alignment methods on 51 pairs involving the CASP14 hard targets together with the maximal TM score (of TM-align).

Blosum62	MRFalign	HHalign	CRFalign (3-State)	CRFalign (5-State)	TM-Align
0.3077	0.3693	0.3905	0.4198	0.4294	0.5973

**Table 6:** Summary of Average TM scores of 300 targets.

	Easy	Medium	Hard	All
Top template from database	0.8902	0.7240	0.4882	0.7860
Best in CRFpred top five	0.8837	0.7050	0.4431	0.7704

## Data Availability

Training and test sets are available from S.J.L. (yeesj123@gmail.com).

## Appendix A

We consider the following input features for the regression trees of the function *F*.

(A) Features for match states:

We assume that the *i*-th position of the template (structure) sequence is aligned (i.e., matched) to the *j*-th position of the target sequence.

(1) Similarity between the HMM-profile of the structure and that of the template, which is calculated as the logarithm with base 2 of the dot product between the HMM-profiles of *s* and *t*. The HMM profiles are obtained from HHblits developed by Söding, et al.

(A1) $$\mathrm{HH}\left(i,j\right)\equiv \log_{2}\left(\sum_{k}^{}{H^{s}}_{i,k}\cdot {H^{t}}_{j,k}\right)$$

where ${H^{s}}_{i,k}$ denotes the *k*-th component of the hmm profile (position specific scoring matrix) at the residue position *i* of the structure template, and ${H^{t}}_{j,k}$ denotes the *k*-th component of the hmm profile at the residue position *j* of the target.

(2) Secondary structure similarity: We employ three-state classification for secondary structures, i.e., *C* (=Coil), *H* (=α-Helix), and *E* (=β-strand). For the template structures *s*, each residue position is assigned by DSSP [31] one of these three states. For the targets, we use the 3-state secondary structure prediction of PSIPRED which gives three values of relative likelihood of secondary structures corresponding to (*C*, *H*, *E*). For a match between the observed secondary structure in 3-state representation at *i*-th position of the template structure and the predicted secondary structure at *j*-th position of the target, the secondary structure similarity is determined as the component value of the predicted secondary structure at *j*-the position of the target corresponding to the observed secondary structure at *i*-th position of the template structure as follows

(A2) $$\mathrm{SS}\left(i,j\right)\equiv \mathrm{SSP}\left(j,k_{i,obs}\right)$$

where $k_{i,obs}$ denotes the observed secondary structure (one of *C*, *H*, or *E*) at the residue position *i* of the template, and $\mathrm{SSP}(i,k_{i,obs})$ denotes the corresponding predicted (PSIPRED) value of the secondary structure at the residue position *j* of the target.

(3) Solvent accessibility: As for the solvent accessibility feature, we use a three-state classification scheme with labels of ’Buried’, ’Intermediate’, and ’Exposed’ states. These are determined by the values of the relative solvent accessibility (RSA) of a specific residue with ranges 0–9% (’Buried’), 9–36% (’Intermediate’), and 36–100% (’Exposed’). For the template structure, the solvent accessibility state is determined by DSSP. Also for the case of target (query) sequence, we employ the program SANN [36] which predicts RSA propensity with 3-state scheme for a sequence with unknown 3D structure.

For a match between the observed RSA (obtained from DSSP) at *i*-th position of the template and the predicted RSA (from SANN) at *j*-th position of the target, the feature value is chosen as

(A3) $$\mathrm{SA}\left(i,j\right)\equiv \mathrm{SANN}\left(j,a_{i,obs}\right)$$

where $a_{i,obs}$ denotes the observed RSA (one of the three states ‘Exposed’, ‘Buried’, or ‘Intermediate’) at the residue position *i* of the template (from DSSP), and $\mathrm{SANN}(j,a_{i,obs})$ denotes the corresponding predicted value of the RSA at the residue position *j* of the target.

(4) BLOSUM matrix, Gonnet matrix, and Kihara matrix: For a match state between *s* at position *i* and *t* at position *j*, we take as additional features the corresponding matrix elements of the BLOSUM62 matrix [35] $B(s_{i},t_{j})$, Gonnet250 matrix [37] $G(s_{i},t_{j})$, and the Kihara matrix [38] $K(s_{i},t_{j})$.

(5) Environmental fitness score:

For the residue position *i* of the template, DSSP provides the secondary structure and the solvent accessibility for the residue. The environmental fitness score for the match between *i*-th residue position of the template and *j*-th residue position of target is obtained from the weighted average of the environmental fitness potential of the template’s local environment state with the position specific frequency matrix (PSFM) $H^{t}\left(j,k\right)$ derived from the HMM profile of the target as follows

(A4) $$\mathrm{Env}\left(i,j\right)\equiv \sum_{k}\phi_{env}\left(k,ss_{i},sa_{i}\right)\cdot H^{t}\left(j,k\right)$$

where $\phi_{env}\left(k,ss_{i},sa_{i}\right)$ denotes the environmental fitness potential of amino acid *k* for the secondary structure state $ss_{i}$ and solvent accessibility state $sa_{i}$ of the template at *i*-th residue position (which was borrowed from the PROSPECT II [39]).

(6) Neighborhood similarity score:

For the match between *i*-th position of the template and *j*-th position of the target, this score measures the similarity between the neighboring residues within a fixed window. Suppose we set the window size $n_{w}\equiv 2*f+1$ with f≥1, then the neighborhood similarity between the template and the target at offset position *k* from (*i*,*j*) is defined as the sum of Pearson correlations of the PSFM, SS and SA at (i+k)-th residue position of the template and the (j+k)-th residue position of the target with k=0, ±1, ±2, ⋯, ±f.

(A5) $$\mathrm{Ns}\left(i,j\right)\equiv \sum_{k=-f}^{k=+f}\left(\mathrm{Corr}\left(H^{s}\left(i+k\right),H^{t}\left(j+k\right)\right)+\mathrm{Corr}\left(SS\left(i+k\right),SS\left(j+k\right)\right)+\mathrm{Corr}\left(SA\left(i+k\right),SA\left(j+k\right)\right)\right).$$

Here Corr(x,y) denotes the Pearson correlation of vectors *x* and *y* with the same length of components which is defined as

(A6) $$\mathrm{Corr}\left(x,y\right)\equiv \frac{\sum_{i=1}^{N}\left(x_{i}-\bar{x}\right)\left(y_{i}-\bar{y}\right)}{\sqrt{\sum_{i=1}^{N}{\left(x_{i}-\bar{x}\right)}^{2}}\sqrt{\sum_{i=1}^{N}{\left(y_{i}-\bar{y}\right)}^{2}}}$$

where $\bar{x}\equiv \sum_{i=1}^{N}x_{i}/N$ is the average value of $x_{i}$, i=1,…,N. As for the size of the neighboring window, we set $n_{w}=9$ (i.e., f=4).

(B) Input features for the Gap states:

(1) Seven-component reduced-profile features derived from the HMM profile based on seven classes of residues:

For a gap state (both at the template and target), the corresponding inserted residue was classified into seven classes as (i) class *I* of hydrophobic and aliphatic residues including Ala, Ile, Leu, and Val. (ii) class II of hydrophobic and aromatic residues including Phe, Trp, and Tyr. (iii) class III of polar residues including Asn, Cys, Gln, Met, Ser, and Thr. (iv) class IV of Acidic charged residues including Asp, and Glu. (v) class *V* of Basic charged residues including Arg, His and Lys. (vi) class VI of the residue Gly. (vii) class VII of the residue Pro.

Suppose there is a gap at position *i* in the template with the corresponding residue at position *j* of the target, one can construct a simple reduced profile by collecting and summing those component values of the PSFM (derived from the HMM profile) of the target residue at *j*-th position, such that those components belonging to the same class are summed over. That is,

(A7) $$C_{7,s}\left(i,j,u\right)\equiv \sum_{k\in u}^{20}H^{t}\left(j,k\right)$$

where ${H^{t}}_{(}j,k)$ denotes the *k*-th component of the HMM profile (position specific scoring matrix) at the residue position *i* of the target. The seven-class index *u* ranges from the class *I* to the class VII as indicated above. Similar method can be applied to the case of a gap in the target.

(2) Secondary structure:

For a gap state at the target (i.e., insertion at the template), the observed secondary structure (from DSSP) of the template at the corresponding inserted residue is used as the secondary structure feature, which is represented in a 3-vector form with the Coil (*C*) state corresponding to (1,0,0), the Helix (*H*) state to (0,1,0) and the Extended Beta (*E*) to (0,0,1). On the other hand, for a gap state at the template (i.e., insertion at the target), the predicted secondary structure propensity with three components (from PSIPRED) of the target at the corresponding inserted residue is used as the secondary structure feature.

(3) Solvent accessibility:

Similarly to the case of secondary structure, for a gap state at the target (i.e., insertion at the template), the observed solvent accessibility in three states (derived from DSSP) of the template at the corresponding inserted residue is used as the input feature. The three states of “Buried” (*B*), “Intermediate” (*I*), and “Exposed” (*E*) are represented as (1,0,0), (0,1,0), and (0,0,1) respectively. On the other hand, for a gap state at the template (i.e., insertion at the target), the predicted solvent accessibility in three states (from SANN) of the target at the corresponding inserted residue is used as the input feature.

(4) Local gap propensity from secondary structure environment:

For a gap state at the target (i.e., insertion at the template), the predicted secondary structure information (from PSIPRED) of the target in the seven neighboring residues (i.e., from −3 to +3 separation from the gap position) is combined to give the Coil (or Loop) propensity as

(A8) $$SS^{g}\left(i,j\right)\equiv \sum_{k=-3}^{+3}\left((SP(j+k,C)-SP(j+k,H))+(SP(j+k,C)-SP(j+k,E))\right)$$

where SP(j+k,C) denotes the component of the predicted secondary structure at the residue position j+k of the target, to be found in a Coil state *C*, SP(j+k,H) the component of the predicted secondary structure to be found in a Helix state *H*, and similarly SP(j+k,E) the component for the Beta strand state *E*.

On the other hand, for a gap state at the template (i.e., insertion at the target), we use similar formula on the neighboring residues for the template except that here, instead of the predicted secondary structure, the observed secondary structure is used, which is represented in a 3-vector form with the Coil (*C*) state corresponding to (1,0,0), the Helix (*H*) state to (0,1,0) and the Extended Beta (*E*) to (0,0,1).

(5) Local gap propensity from solvent accessibility environment:

For a gap state at the target (i.e., insertion at the template), the predicted solvent accessibility information (from SANN) of the target in the seven neighboring residues (i.e., from −3 to +3 separation from the gap position) is combined to give the Coil (or Loop) propensity as

(A9) $${\mathrm{SA}}^{g}\left(i,j\right)\equiv \sum_{k=-3}^{+3}\left(0.2*\mathrm{SANN}(j+k,I)+0.7*\mathrm{SANN}(j+k,E)\right)$$

where SANN(j+k,I) denotes the component of the predicted solvent accessibility at the residue position j+k of the target, to be found in an “Intermediate” state *I*, SANN(j+k,E) the component of the solvent accessibility to be found in an “Exposed” state *E*. Hence we put more weight on the exposed residues than buried or intermediate residues.

Similarly for a gap state at the template (i.e., insertion at the target), we use similar formula on the neighboring residues for the template except that here, instead of the predicted solvent accessibility, the observed solvent accessibility (in normalized 3-vector form) is used. Therefore, the summand in the above formula becomes 0.2 for the state “Intermediate” and 0.7 for the “Exposed” state.

(6) Additional features for indicators of the end position of the sequences:

In addition to the above input features for the gap states, we also use separate indicators for the end positions of each of the two sequences for special treatment of end gaps. That is, we define

(A10) $$\mathrm{End}\left(i,j\right)\equiv \delta_{i}+\delta_{j}$$

where $\delta_{i}$ ($\delta_{j}$) =1 if *i* (*j*) is at the beginning (N-terminal) or at the end (C-terminal) of the sequence and 0 otherwise.
